# What treatment outcomes matter in adolescent depression? A Q-study of priority profiles among mental health practitioners in the UK and Chile

**DOI:** 10.1007/s00787-023-02140-9

**Published:** 2023-01-31

**Authors:** Karolin Rose Krause, Ana Calderón, Victor Gomez Pino, Julian Edbrooke-Childs, Bettina Moltrecht, Miranda Wolpert

**Affiliations:** 1 Research Department for Clinical, Educational and Health Psychology, University College London, Gower Street, Bloomsbury, London, WC1E 6BT UK; 2 Cundill Centre for Child and Youth Depression, Centre for Addiction and Mental Health (CAMH), Toronto, Canada; 3 Facultad de Psicología y Humanidades, Universidad San Sebastián, Sede Santiago, Chile; 4 Facultad de Medicina, Departamento de Psiquiatría Norte, Hospital Clínico, Clínica Psiquiátrica Universitaria, Universidad de Chile, Avenida La Paz 1003, Recoleta, Chile; 5 Evidence-Based Practice Unit, Anna Freud National Centre for Children and Families, 4–8 Rodney Street, London, N1 9JH UK; 6 Centre for Longitudinal Studies, University College London, 55-59 Gordon Square, London, WC1H 0NU UK; 7 Wellcome Trust, 215 Euston Rd, Bloomsbury, London, NW1 2BE UK

**Keywords:** Adolescents, Depression, Psychotherapy outcome research, Q-methodology, Q-study, Clinician perceptions, Outcome priorities, Cross-cultural research

## Abstract

Evidence-based and person-centred care requires the measurement of treatment outcomes that matter to youth and mental health practitioners. Priorities, however, may vary not just between but also within stakeholder groups. This study used Q-methodology to explore differences in outcome priorities among mental health practitioners from two countries in relation to youth depression. Practitioners from the United Kingdom (UK) (*n* = 27) and Chile (*n* = 15) sorted 35 outcome descriptions by importance and completed brief semi-structured interviews about their sorting rationale. By-person principal component analysis (PCA) served to identify distinct priority profiles within each country sample; second-order PCA examined whether these profiles could be further reduced into cross-cultural “super profiles”. We identified three UK outcome priority profiles (*Reduced symptoms and enhanced well-being; improved individual coping and self-management; improved family coping and support*), and two Chilean profiles (*Strengthened identity and enhanced insight; symptom reduction and self-management*). These could be further reduced into two cross-cultural super profiles: one prioritized outcomes related to reduced depressive symptoms and enhanced well-being; the other prioritized outcomes related to improved resilience resources within youth and families. A practitioner focus on symptom reduction aligns with a long-standing focus on symptomatic change in youth depression treatment studies, and with recent measurement recommendations. Less data and guidance are available to those practitioners who prioritize resilience outcomes. To raise the chances that such practitioners will engage in evidence-based practice and measurement-based care, measurement guidance for a broader set of outcomes may be needed.

**Supplementary Information:**

The online version contains supplementary material available at 10.1007/s00787-023-02140-9.

## Introduction

Depression is a common, serious mental health condition in adolescents that is associated with increased rates of recurrence, suicide, other mental and physical health conditions, and adverse socio-economic outcomes across the life span [1–5]. Early and effective intervention is critical. Yet, in clinical trials and routine specialist care, around 40% of youth leave treatment without showing meaningful improvement [6, 7].

Mechanisms that may improve care effectiveness include evidence-based practice [8], measurement-based care [9–11] and person-centred care [12, 13]. Evidence-based practice involves the integration of the best available research evidence with clinical expertise and service user values to inform clinical and policy decision-making [14]. Measurement-based care involves collecting outcome data from individual service users and using these data to inform treatment planning [11, 15]. Randomized control trials show significant positive effects of measurement-based care on treatment outcomes of adults receiving psychotherapy [e.g., 11, 16–19], and antidepressants [20], though the evidence base is less developed for youth [21]. Person-centred care encourages the active engagement of service users in care management, for example through shared decision-making about treatment options [13].

The three approaches go hand-in-hand where mental health practitioners and service users jointly consider data from research studies or measurement-based care, and use shared decision-making to agree next steps for treatment in line with service user preferences [22, 23]. In this context, it is critical that the outcomes measured in research and measurement-based care are meaningful to service users and practitioners. Uptake of measurement-based care in youth mental health care has been modest, with one barrier being practitioner skepticism about the ability of commonly used outcome measures to adequately capture subtle change, complex trajectories, or individual treatment goals [24–27].

In recent years, interest in what constitutes a meaningful outcome for youth depression has grown. Two systematic reviews of outcomes reporting in clinical trials have shown that these trials predominantly report on depressive symptoms, and rarely cover domains such as coping skills, quality of life, relationships, parental support, or personal growth [28, 29]. Qualitative studies that examined outcomes discussed by youth and clinicians following treatment for depression suggest that while both groups consider symptom severity an important outcome, they also value some of the above-mentioned, rarely measured outcomes [30, 31]. An expert working group of researchers, clinicians, youth and family representatives convened by the International Consortium for Health Outcomes Measurement (ICHOM) recently reached the consensus recommendation that symptom severity, functioning, and suicidality should be assessed as a minimum for all youth who receive depression care [32]. A similar initiative focused on recommendations for youth depression clinical trials is underway [33].

Less attention has been given to how outcome priorities vary within and between stakeholder groups. Heterogeneity must, however, be considered if outcome measurement is to be truly person-centred, by catering to service users with potentially different priorities, as well as practitioners with different types of training and outlook. It has been suggested that judgments of what constitutes a “good outcome” are the product of values and norms that may vary across cultural contexts, or as a function of the theoretical orientation of clinical training [34–38]. Depending on their preferred “theory of therapeutic action” [39], practitioners may target different outcomes based on different assumptions about the ideal sequencing of change during therapy [37]. For example, in qualitative interviews following treatment for adolescent depression, psychoanalytic psychotherapists more commonly discussed change related to young people’s sense of self, while cognitive-behavioural therapists more frequently discussed changes in cognition and behaviour [31].

A recent study using Q-methodology (i.e., a card sorting exercise and factorial analysis to identify sorting patterns among participants) identified four outcome priority profiles among youth aged 16–21 years with experience of depression. While all profiles prioritized outcomes related to improved mood and reduced anhedonia, the youth differed in the importance assigned to the acquisition of coping skills, to the processing of past and current experiences, and to reduced functional impairment [40]. Similar insights about heterogeneity in outcome priorities are lacking for practitioners treating youth for depression, though Q-methodological studies have explored practitioners’ perspectives on recovery from adult psychosis [41], and adult borderline personality disorder [42]. In addition, to our knowledge, no study has yet explored linkages between cultural context and outcome priorities among mental health practitioners. This study aimed to address these gaps by identifying profiles of outcome priorities for the treatment of youth depression in a purposive cross-cultural sample of mental health practitioners from the United Kingdom (UK) and Chile; and by exploring intercultural differences in priorities.

## Method

### Overall design considerations

We chose Q-methodology to examine outcome priority profiles because this method combines quantitative and qualitative research aspects in the study of subjective viewpoints and preference profiles [43–45]. In a Q-methodological study, an item set (e.g., statements printed on cards) representing the discourse on a topic of interest is sorted by participants according to a ranking scheme (e.g., by perceived importance or by the participant’s level of agreement with each statement). Patterns of similarity and difference in how participants sorted the items are then analyzed using inverted or “by-person” factor analysis, where factors are based on the correlations between the participants’ sorts (called *Q-sorts*), rather than the correlation between individual items or variables from the item set. Through this process, the variance across all participant Q-sorts is reduced into a smaller number of factors that represent distinctive viewpoints [43–45].

We selected the UK and Chile for this cross-cultural comparison, because youth depression is common in both contexts [46, 47], and both are high-income countries with comparatively well-developed mental health systems. The two countries have nonetheless been described as adhering to different cultural spheres [48]. A brief comparison of the two contexts is provided in Panel 1.

Panel 1. Brief comparison of the UK and Chilean contexts In the UK, prevalence rates for depressive disorders are 2.7% among 11–16 year-olds and 4.7% among 17–19-year-olds [46]. In Chile, the 12-month prevalence rate for unipolar depression among 12–18-year-olds has been estimated at 7% [47]. In the UK, mental health care is part of the universal healthcare system funded through general taxation [49]. Chile employs a two-tiered model with a larger private sector, and a public health system that covers around 72% of the population [50, 51]. In the UK, individual cognitive behavioral therapy (CBT) is recommended as the first-choice treatment for moderate to severe depression in youth by the UK’s National Institute for Health and Care Excellence’s (NICE) clinical practice guidelines [52]. Alternative options are Interpersonal Psychotherapy, systemic or attachment-based family therapy, brief psychosocial intervention, or psychodynamic psychotherapy.

Chilean clinical practice guidelines recommend CBT, interpersonal therapy, systemic therapy and behavioural activation for the treatment of adult depression, without specifying specific suitable approaches for youth [53]. Eclectic, psychodynamic, cognitive-behavioural and systemic perspectives have been described as the common approaches in Chile [54]. Culturally, the UK is considered part of the Anglo-Saxon cultural sphere with an emphasis on secular and post-materialist values (e.g., importance of tolerance, ecology, freedom of choice, life satisfaction); Chile is considered part of the Latin-American cultural cluster with greater emphasis on traditional, Roman Catholic values (e.g., importance of family, faith, and respect for authority) [48].

### Participants

Q-methodological studies seek to identify viewpoints and to describe them in some depth; they do not typically aim to assert the prevalence or representativeness of these viewpoints [41]. Hence, Q-methodological studies often employ a qualitative sampling approach with between 20 and 50 purposively sampled participants. Based on inverting sample size guidelines for traditional factor analysis, the number of Q-study participants should not exceed the number of items to sort [55, 56]. As our study used a 35-item Q-set (see below), we aimed to recruit a maximum sample of 30 participants per country.

We anticipated that outcome priorities might vary by profession, theoretical training, and work setting, and aimed for diversity in these regards [31, 34, 36]. Any practitioner providing mental health support to adolescents with depression was eligible. In the UK, we advertised recruitment calls through flyers and posters that were shared with and by youth mental health charities and via social media. In Chile, practitioners were recruited from private university mental health services. In both countries, we used a combination of convenience and snowball sampling [58].

### Procedure

The Q-set used in the present study was developed through a multi-stage process involving stakeholder workshops, a systematic review of youth depression treatment studies [28]; and a qualitative analysis of youth and clinicians’ outcome narratives following treatment for depression [31]. A detailed description of this process has been published elsewhere [40]. The final Q-set consisted of 35 cards, each of which carried an outcome description. The outcomes covered the domains of symptoms, self-management, functioning, personal growth, relationships, therapeutic space, youth well-being, and parental support and well-being, with four to five outcomes included per domain. The Q-set was translated from English into Spanish, and back-translated into English by an independent translator fluent in both languages to ensure that the original meaning had been preserved [59]. The final Q-set is reported in Table 3 (English) and in Supplement 1 (Spanish).

**Fig. 1:**
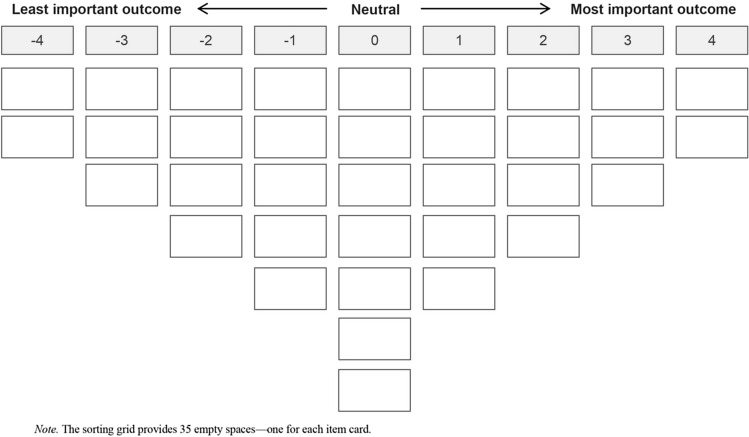
Sorting grid used by participants

Participants were asked to rank order the 35 outcomes according to an inverted bell shape using a sorting grid with a 9-point scale of importance (from + 4 *most important,* to − 4 *least important,* see Fig. 1). After the sorting, participants completed a brief demographic questionnaire, and in a brief semi-structured interview (recorded and transcribed verbatim) were asked to explain their sorting rationale and suggest any missing outcomes [60]. The research tasks were completed in English or Spanish. Practitioners completed the card sorting task at individual appointments or as part of professional workshops. Post-sort interviews were conducted privately in person or over the telephone.

### Data analysis

Q-sort data from the UK and Chilean samples were analyzed separately in the first instance. We used by-person principal component analysis (PCA) via the PQMethod analysis software [61] to identify highly correlated practitioner Q-sorts within each sample. The most suitable principal component solution was determined by examining the scree plot of Eigenvalues, the shared variance explained, the number of Q-sorts loading significantly (i.e. at *p* < 0.01) on a single component, and the correlation between components. Varimax rotation and adjustments via hand rotation aimed to maximize the number of significantly loading Q-sorts [55]. An *ideal–typical Q-sort* was generated for each extracted component (i.e., outcome priority profile) by averaging the outcome rankings across all significantly loading Q-sorts, using the correlation coefficients as weights [62]. The ideal–typical Q-sort shows the importance that would be assigned to each outcome by an exemplary representative with a maximal loading on the relevant component/priority profile [55]. The brief semi-structured interviews were analyzed in their original language using qualitative content analysis [63].

The results section presents narrative interpretations of each priority profile with cross-references to the exemplary Q-sort (item numbers and ranks are provided in parentheses, e.g., #3, + 3), and to the interview data. Spanish quotes were translated into English by one author and reviewed by a second author for inclusion in this report.

To examine whether the country-specific priority profiles aggregated into a smaller number of cross-cultural “super profiles”, we performed a second-order principal component analysis [64]. This followed the same statistical procedure detailed above, but with the ideal–typical Q-sorts rather than the actual Q-sorts produced by participants as raw data [55]. Exploratory descriptive analysis examined whether there appeared to be any association between practitioner characteristics and super profiles.

### Ethical approval

This study was approved by the ethics review board (REB) of University College London in March 2018 (UCL REC REF: 10,567/002), and by the REBs of the participating Chilean institutions between June and July 2018. All participants provided informed written consent. To ensure confidentiality, all interview data have been deidentified. This research has thus been performed in accordance with the ethical standards laid down in the 1964 Declaration of Helsinki and its later amendments.

## Results

### UK practitioner sample

#### Participant characteristics

In the UK, 30 practitioners completed the Q-sort. Three were excluded from the analysis due to having their residence outside of the UK, yielding a final sample of 27. The majority were female (*n* = 17; 63%); the mean age was 42 years (see Table 1). Participants had an average of 15 years of experience working in child and adolescent mental health (CAMH), and identified as clinical psychologists (*n* = 9), psychotherapists (*n* = 5), psychiatrists (*n* = 5), or other practitioners (e.g., mental health nurses, support workers). Most practitioners stated working in specialist outpatient services (*n* = 11; 41%) or mental health charities (*n* = 8; 30%). Commonly used treatment approaches included cognitive-behavioural therapy (CBT, *n* = 14; 52%), systemic therapy (*n* = 12; 44%), and psychoanalytic/psychodynamic approaches (*n* = 7; 26%).

**Table 1:** Q-sort participants’ demographic characteristics by study location

Variable	UK	Chile
	*N* (%)	*N* (%)
*N*	27 (100%)	15 (100%)
Gender		
Male	10 (37%)	≤ 3 (20%) ^a^
Female	17 (63%)	≥ 12 (80%) ^a^
Age (in years)		
Mean (SD)	42.4 (11.5)	41.5 (10.2)
Range	22–65	23–62
Profession		
Clinical psychologist	9 (33%)	9 (60%)
Psychotherapist	5 (19%)	4 (27%)
Psychiatrist	5 (19%)	≤ 3 (20%) ^a^
Other^b^	8 (30%)	–
Professional experience in CAMH (in years)		
Mean (SD)	15.4 (9.7)	15.5 (8.0)
Range	1–37	1–28
Professional setting		
Specialist CAMH outpatient service	11 (41%)	–
Mental health charity	8 (30%)	–
Specialist CAMH inpatient service	≤ 3 (11%)^a^	–
School or university	≤ 3 (11%)^a^	15 (100%)
Other setting^c^	≤ 3 (11%)^a^	–
Psychotherapeutic approach		
Cognitive behavioural^d^	14 (52%)	≤ 3 (20%) ^a^
Systemic	12 (44%)	9 (60%)
Psychoanalytic/dynamic^e^	7 (26%)	6 (40%)
Pharmacological	6 (22%)	≤ 3 (20%) ^a^
Interpersonal^f^	5 (17%)	–
Other^g^	9 (33%)	4 (15%)

^a^Cell sample sizes below *n* = 3 are suppressed due to confidentiality considerations

^b^Other professions included mental health nurses, service commissioners, social workers, and mental health support workers

^c^Other settings include outreach teams and clinical commissioning groups

^d^Cognitive behavioural includes behavioural activation, problem solving, dialectical behaviour therapy (DBT), trauma-focused CBT

^e^Includes psychoanalytic and psychodynamic psychotherapy, as well as mentalization-based therapy

^f^Interpersonal approaches include interpersonal psychotherapy and social skills training

^g^In the UK, other psychotherapeutic approaches included supportive counselling, psychoeducation, advice & signposting, guided self-help, crisis support, direct inpatient care, parent and group work; in Chile, other approaches included existential/experiential approaches and strategic therapy

### Principal component solution

We extracted three principal components (A_UK_, B_UK_ and C_UK_) that explained 49.6% of the common variance in UK Q-sorts. Varimax rotation and adjustments via hand rotation (i.e., rotating component axes 1 and 2 by + 2 degrees; axes 2 and 3 by + 2 degrees; and axes 1 and 3 by 9 + degrees) were used to enhance the clarity of the component solution, which accounted for 24 of the 27 Q-sorts. Two Q-sorts did not load significantly on any extracted component, and one loaded on more than one component (i.e., was confounded). These Q-sorts were excluded from the generation of the ideal–typical Q-sorts [55]. Inter-component correlations ranged from 0.11 to 0.32 but did not reach statistical significance, suggesting the components represent distinct profiles. Table 2 shows the component loadings for each UK Q-sort. Table 3 shows the ideal–typical Q-sorts. In the remainder of this report, we will refer to the extracted components as “priority profiles”.

**Table 2:** Rotated component loadings following country-specific PCA

Component loadings						
Outcome priority profile	Practitioner Q-sort	UK			Chile	
UK sample		A_UK + /UK −_	B_UK_	C_UK_		
Reduced symptoms and enhanced well-being	P1—Clinical psychologist	**0.75**	0.15	0.36		
	P2—Psychiatrist	**0.73**	− 0.21	0.27		
	P3—Psychiatrist	**0.71**	0.07	0.28		
	P4—Psychiatrist	**0.67**	0.29	0.32		
	P5—Clinical psychologist	**0.66**	0.35	− 0.13		
	P6—Clinical psychologist	**0.62**	− 0.04	0.09		
	P7—Clinical psychologist	**0.57**	0.24	0.03		
	P8—Clinical psychologist	**0.52**	− 0.02	0.14		
	P9—Clinical psychologist	**0.48**	0.03	− 0.16		
	P10—Psychiatrist	**0.44**	0.25	− 0.09		
	P11—Psychiatrist	**− 0.52**	− 0.27	0.33		
	P12—Other practitioner	**− 0.66**	0.32	0.38		
Improved individual coping and self-management	P13—Psychotherapist	0.08	**0.89**	0.06		
	P14—Clinical psychologist	0.13	**0.83**	− 0.04		
	P15—Nurse	0.18	**0.78**	0.04		
	P16—Nurse	0.06	**0.62**	0.02		
	P17—Other practitioner	0.12	**0.61**	0.42		
	P18—Nurse	0.25	**0.57**	0.28		
	P19—Psychotherapist	0.20	**0.53**	0.35		
Improved family coping and support	P20—Psychotherapist	− 0.40	− 0.08	**0.67**		
	P21—Other practitioner	0.29	0.31	**0.65**		
	P22—Other practitioner	0.04	0.23	**0.61**		
	P23—Psychotherapist	0.31	0.02	**0.61**		
	P24—Clinical Psychologist	0.13	0.23	**0.51**		
Not assigned	P25—Psychotherapist^a^	0.38	0.29	0.20		
	P26—Other practitioner^a^	− 0.03	0.21	− 0.24		
	P27—Clinical Psychologist^b^	0.49	0.09	0.67		
% of the variance explained		20.4	16.2	13.0		
Composite reliability coefficient		0.98	0.97	0.95		
Chilean sample					A_CL_	B_CL_
Strengthened identity and enhanced insight	P28—Clinical psychologist				**0.88**	− 0.02
	P29—Clinical psychologist				**0.74**	− 0.09
	P30—Clinical psychologist				**0.70**	− 0.01
	P31—Clinical psychologist				**0.69**	− 0.26
	P32—Clinical psychologist				**0.59**	0.43
	P33—Psychotherapist				**0.57**	0.14
	P34—Clinical psychologist				**0.57**	0.12
	P35—Psychiatrist				**0.56**	0.11
	P36—Psychotherapist				**0.50**	0.35
Reduced symptoms and enhanced well-being	P37—Clinical psychologist				0.08	**0.82**
	P38—Psychiatrist				− 0.27	**0.72**
	P39—Clinical psychologist				0.33	**0.68**
	P40—Psychotherapist				0.43	**0.65**
Not assigned	P41—Psychotherapist^a^				0.12	0.38
	P42—Clinical psychologist^b^				0.57	0.68
% of the variance explained					30.0	21.0
Composite reliability coefficient					0.97	0.94

Component loadings printed in bold were significant at the *p* < 0.01 level (i.e., loadings equal to or above 0.44). The corresponding Q-sort contributed to computing the ideal–typical Q-sort for this component

^a^These Q-sorts did not load significantly on any of the four components and were excluded from the computation of the ideal–typical Q-sorts

^b^This Q-sort had significant loadings on two components and was therefore excluded from the computation of the ideal–typical Q-sorts

**Table 3:** Ideal–typical Q-sorts by priority profile

# Q-sort item		Ideal–typical item ranks				
		UK			Chile	
		A + _UK_	B_UK_	C_UK_	A_CL_	B_CL_
Symptoms						
1	Being less angry and not losing their temper as much	1	− 2	0	− 2	2
2	Feeling less down and depressed	3	2	− 2	1	4
3	Feeling happier and enjoying things more	4	2	− 1	2	4
4	Feeling more loved	1	− 3	1	0	2
5	Engaging less in behaviour that can be harmful	3	3	3	2	3
Self-management						
6	Being more active and engaged in things	1	− 1	2	− 1	− 1
7	Knowing ways to cope with their emotions	3	4	2	0	2
8	Having a better understanding of their feelings and thoughts	0	4	4	3	0
9	Being able to challenge negative thoughts and approach situations differently	− 1	3	1	1	2
Functioning						
10	Being better able to get things done (e.g., concentrate, be organized)	2	− 3	0	− 2	− 3
11	Being able to do the same things other adolescents do	0	− 2	− 4	− 4	0
12	Working more effectively in school (e.g., being more motivated and focused)	− 1	− 2	− 1	− 3	− 2
13	Attending school more regularly	− 2	− 1	2	− 3	1
14	Being more sociable and better able to be around other people	2	− 2	0	− 1	0
Personal growth						
15	Feeling more confident	2	0	0	4	1
16	Being better able to stand up for their needs and opinions	− 1	− 1	1	0	− 1
17	Being more independent and able to take responsibility for their life	0	0	0	1	− 4
18	Being able to make sense of things that have happened in the past, or are still happening	0	1	− 1	3	− 2
19	Having a better sense of who they are and how to be themselves around others	− 1	0	3	4	− 3
Relationships						
20	Feeling more able to talk about their feelings and thoughts	0	2	0	3	0
21	Getting on better with their family	1	0	1	0	0
22	Getting on better with their friends or having made new friends	1	− 1	0	− 2	1
23	Getting on better with their peers in school (e.g., not feeling bullied)	0	− 1	1	− 1	− 1
Therapeutic space						
24	Having a space where someone listens and cares about them	− 2	1	− 3	2	1
25	Having a space where they can let out my feelings	− 4	0	− 2	1	0
26	Having a space where they can talk about anything without being judged	− 2	1	− 3	1	− 2
27	Having a space to reflect and think about things differently	− 3	3	− 3	2	− 1
Youth Well-being						
28	Having greater peace of mind (e.g., feeling calmer, more balanced)	0	0	− 2	− 1	3
29	Feeling more optimistic and positive about life and the future	4	2	− 1	0	3
30	Feeling physically healthier	− 3	− 4	− 1	− 2	− 2
31	Being able to make plans for the future and have goals	2	1	2	0	1
Parental support and well-being						
32	Parents feeling happier and less stressed and worried	− 3	− 3	− 4	− 3	− 4
33	Parents having a better understanding of them and their difficulties	− 2	1	3	− 1	− 1
34	Parents feeling more able to support them	− 1	0	4	0	0
35	Parents feeling less guilty	− 4	− 4	− 2	− 4	− 3

Reading Table 3 by columns conveys the rankings assigned to all 35 items for a given exemplary Q-sort (from rank + 4 through rank -4); Reading the table by row allows for comparing rankings for one specific item across components

### Consensus outcomes

Several outcomes were ranked similarly by all three UK-based profiles, with no significant difference at the *p* > 0.1 level. All profiles prioritized reductions in risky behaviour and self-harm (#5, + 3), with practitioners emphasizing the importance of keeping youth safe. Being able to make plans for the future and have goals was consistently ranked as somewhat important (#31, + 1 or + 2); and youth getting on better with their family was ranked as neutral to important (#21, 0 or + 1). Practitioners consistently ranked improved parental well-being (#32, − 3 or − 4) among the least important outcomes, often suggesting that achieving this outcome was beyond their remit.

### Outcome priority profile *A*_*UK*_: reduced symptoms and enhanced well-being

Profile A_UK_ explains 20.4% of the common variance in UK Q-sorts, representing 12 UK-based practitioners. Ten of these practitioners loaded positively on this component and two loaded negatively. A component that consists of Q-sorts with significant positive and negative loadings conveys two opposed viewpoints, associated with ideal–typical Q-sorts that are the mirror image of one another [65]. We will focus on describing the major profile A_UK+_, and provide a brief account of the outcomes prioritized by Profile A_UK−_.

The most important outcomes for Profile A_UK+_ were reductions in core depressive symptoms like low mood (#2, + 3) and anhedonia (#3, + 4), as well as self-harm (#5, + 3). Practitioners also emphasized the importance of restoring a sense of optimism (# 29, + 4) and the ability to make plans and goals for the future (#31, + 2):

“The ability to taste and enjoy pleasure—that seems, to me, fantastic and important and […] with the sort of future facing optimism, there’s hope, there’s agency.” (P3, psychiatrist, UK)

“Feeling less depressed and feeling happier, to me, are the opposite to depression and if I’m helping someone with depression then almost by definition, that’s what we’re going to be heading for.” (P1, clinical psychologist, UK)

Improved coping skills (#7, + 3) were considered important, but changes in youth’s ability to understand, manage, and convey emotions (#8, 0; #9, − 1; #20, 0), and personal growth (e.g., gaining a stronger sense of self, #19, − 1) were ranked lower than by other profiles. Profile A_UK+_ practitioners tended to deprioritize process outcomes related to youth having a space where they could express and reflect on their feelings and thoughts (#25, − 4; #27, 3), as well as outcomes related to improved parental well-being and support (# 32–35; ranks – 1 to − 4).

The two practitioners associated with the opposite profile A_UK−_ deprioritized reductions in core depressive symptoms, which they described as only an initial step in a longer journey towards recovery and enhanced independence. Profile A_UK−_ practitioners considered it important to empower youth and parents to be active agents of this journey, by enhancing youth assertiveness, reducing parental guilt, and improving parental support.

On average, practitioners in profile A_UK+_ had 19.0 years (SD = 11.2) of professional experience in CAMH. Six identified as clinical psychologists and four as psychiatrists. Commonly used treatments included CBT (*n* = 7), systemic therapy (*n* = 6), and psychopharmacological treatment (*n* = 3). Characteristics of the two practitioners in profile A_UK-_ are suppressed to protect their anonymity.

### Outcome priority profile B_*UK*_: improved individual coping and self-management

Profile B_UK_ explained 16.2% of the common variance, representing seven UK-based practitioners who focused specifically on improving youth’s individual coping and self-management skills. These practitioners considered difficult emotions an inherent part of life, and suggested that living a life free of any depressive symptoms might be unrealistic for some youth. Instead of reductions in depressive symptoms (#2 and #3, + 2), they prioritized outcomes related to youth learning coping skills (#7, + 4), gaining a better understanding of their feelings and thoughts (#8, + 4), and learning to challenge negative thoughts (#9, + 3).

“Emotions are normal and some people have more extreme emotions than others, dependent on what your experience in life has been. […] With adolescents who have got high levels of need, it’s not necessarily always about … my approach has never always been about making those feelings or emotions go away, it’s about knowing better ways how to manage them day-to-day.” (P18, nurse, UK)

Contrary to other UK profiles, practitioners in Profile B_UK_ prioritized youth having a space to reflect and think about things differently (#27, + 3). Some explained that they aimed to eventually help youth internalize the reflective space offered by therapy, and to build self-help skills, in order to become more resilient in the longer term:

“I think it’s around creating skills in young people, rather than treating them.” (P16, nurse, UK)

“If they’re able to have a space to reflect, to have a better understanding of their feelings and thoughts, then gradually, even without the treatment, they would hopefully continue to improve and grow and develop as people.” (P13, psychotherapist, UK)

Improved peer and school functioning (#10 to #14, − 1 to − 3) was deprioritized, with practitioners explaining that this was likely to occur naturally through more successful coping.

Three practitioners in profile B_UK_ identified as nurses, three as clinical psychologists or psychotherapists, and one as another professional. Practitioners were mostly based at outpatient services, with 10 years of professional experience in CAMH on average (SD = 2.4). All treatment approaches listed in Table 1 were represented.

### Outcome priority profile *C*_UK_: improved family coping and support

Profile C_UK_ explained 13.0% of the common variance and represented five UK-based practitioners. These practitioners converged with Profile B_UK_ on the notion that ups and downs are a feature of life, especially during adolescence, and that becoming symptom-free (#2 and #3, − 1 or − 2) and having greater peace of mind (#28, − 2) might be unrealistic for some youth. But rather than focusing on individual skills and resilience, this profile prioritized parental support (#33 and #34, + 3 or + 4), and clarifying the youth’s role within the family (#19, + 3).

“If the relationship with the parent, the family improves, that often has more impact than anything that’s going on in the room. This is the critical thing for me, how the family can construct a young person’s depression in a way that’s non-critical and supportive and caring.” (P27, clinical psychologist, UK)

Contrary to profile B_UK_, these practitioners deprioritized procedural outcomes related to the therapeutic space (#24 to 27, − 2 or − 3), suggesting that therapy should help create a safe and non-judgmental space *within the family*.

On average, practitioners in profile C_UK_ had 8.5 years (SD = 5.8) of experience working in CAMH. They included clinical psychologists, psychotherapists, and other professionals. Treatment approaches included CBT, systemic therapy, psychoanalytic or psychodynamic approaches, and psychoeducation or guidance.

### Chilean practitioner sample

#### Participant characteristics

Due to administrative delays in the ethical approval process, only 15 Chilean practitioners could be recruited into the study. The majority were female. Ages ranged from 23 to 62 years (mean = 42 years). As shown in Table 1, most practitioners (*n* = 9; 60%) identified as clinical psychologists, some as psychotherapists (*n* = 4; 27%), and a minority as psychiatrists (≤ *n* = 3; ≤ 20%) Professional experience in CAMH ranged from 1 to 28 years, with a mean of 16 years (SD = 8.0 years). The most commonly used treatment approach was systemic therapy (*n* = 9; 60%), followed by psychodynamic or psychoanalytic psychotherapy (*n* = 6; 40%).

### Principal component solution

By-person PCA with Varimax rotation and hand adjustment (i.e., rotation of axes 1 and 2 by 8 degrees) identified two Chilean outcome priority profiles that explained 51% of the common variance in Q-sorts. Inter-component correlation was low (*r* = 0.19). Two Q-sorts were excluded from further analysis, because one did not load significantly on any of the two components, and one was confounded. The rotated component matrix is shown in Table 2. The ideal–typical Q-sorts for each priority profile are shown in Table 3.

### Consensus outcomes

Both Chilean priority profiles considered it important that youth engage less in self-harm or risk-taking behaviour (#5, + 2 or + 3). Similarly, feeling happier and enjoying things more (#3) was considered important by both profiles, though more by Profile B_CL_ (+ 4) than by profile A_CL_ (+ 2). Both profiles deprioritized reduced parental guilt and enhanced parental well-being (#32, #35: − 3 or − 4). Practitioners considered parental guilt a complex issue: for parents who had disengaged from their children’s lives, developing feelings of guilt could form a catalyst for change, while other parents might feel excessively guilty. In general, practitioners suggested that therapy outcomes should centre around the youth’s well-being and ability to negotiate support within their families.

### Outcome priority profile *A*_CL_: strengthened identity and enhanced insight

Profile A_CL_ explained 30.0% of the common variance in Chilean Q-sorts, representing nine practitioners. This profile considered that change had to unfold from the inside out, with the most important outcomes centred within the self. Highly ranked outcomes included a stronger sense of self and ability for youth to be genuinely themselves around other people (#19, + 4), increased confidence (#15, + 4), youth having an improved understanding of their feelings and thoughts (#8, + 3), youth being able to make sense of past or current experiences (#18, + 3), and youth feeling able to talk more openly about feelings and thoughts (#20, + 3). Practitioners described adolescence as a period of identity searching, marked by multiple pressures at home, school, and within the peer group. They considered that developing a balanced and confident self-image and learning to tune into one’s feelings would help youth become more resilient.

“For me change always occurs from the inside out […] the most important thing is that there tends to be a relationship between internal change which then leads to this external change, such as getting along better with others, doing well in school, getting the parents involved and not feeling guilty, stressed, or anything, but first, the idea is that the patient can get to know themselves and from there, work on all these emotions.” (P31, clinical psychologist, Chile)

These practitioners deprioritized the functioning-focused outcome statement “*being able to do the same things other adolescents do”* (#11, − 4), suggesting that it pathologized difference and overvalued conformity. Some practitioners expressed concern about parents and educators overemphasizing performance, and suggested that academic (#12 and 13, − 3), executive (#11, − 2) and social functioning (#14, − 1) would improve naturally as a result of intrapsychic change, if youth were allowed to recover at their own pace.

“Among the least important priorities for me is that this teenager who is having a hard time can do the same thing that all adolescents do […] I can’t assume that in and of itself … since there’s not one teenager that’s the same as another … to standardize them or ask them not to be angry at the world.” (P30, clinical psychologist, Chile)

“At least here [in Chile] everything is always linked to education, going to school, performance, performance. For me, I know it tends to be a concern for parents, it tends to be a concern for teachers, or school psychologists, but for me it is one of the least important things, as long as they feel good about themselves, we can work more slowly.” (P31, clinical psychologist, Chile)

Practitioners in profile A_CL_ had, on average, 13.6 years (SD = 9.7) of professional experience in CAMH and included primarily clinical psychologists with a minority identifying as psychotherapists or psychiatrists. The most common therapeutic approaches were psychodynamic/psychoanalytic, systemic, and existential approaches.

### Outcome priority profile *B*_CL_: symptom reduction and self-management

Profile B_CL_ explained 21.0% of the common variance, representing four Chilean practitioners. This profile prioritized outcomes related to reduced depressive symptoms and enhanced well-being, closely reflecting Profile A_UK+_. The highest ranked outcomes related to feeling less depressed (#2, + 4) and better able to experience joy (#3, + 4), reductions in risky behaviour or self-harm (#5, + 3), improved optimism (#29, + 3), and greater peace of mind (#28, + 3). Practitioners considered that depressive symptoms were usually the primary reason for help-seeking or referral, with many clients defining treatment goals around wishing to feel happier. Contrary to Chilean profile A_CL_, these practitioners deprioritized outcomes related to gaining independence (#17, − 4), developing a stronger sense of self (#19, − 3), and making sense of past experiences (#18, − 2), which they described as possible but not necessary avenues for change.

Practitioners in profile B_CL_ had 17.8 years of professional experience, on average, in CAMH (SD = 6.9). They included clinical psychologists, psychotherapists, and psychiatrists. The treatment approaches most frequently used were systemic therapy, psychopharmacological treatment, CBT, and strategic therapy.

### Second-order principal component analysis—practitioners in the UK and Chile

After entering the ideal–typical Q-sorts for the country-specific profiles into a second-order principal component analysis, a two-component solution provided the best fit, accounting for 72.0% of the common variance in ideal–typical Q-sorts (Table 4). The second-order components were moderately correlated (*r* = 0.31). The first “super profile” represented UK profile A_UK+_ and Chilean profile B_CL_, with their common focus on *reduced symptoms and enhanced well-being*. The second represented the Chilean profile A_CL_ (*strengthened identity and enhanced insight*)*,* the UK profiles B_UK_ (*improved individual coping and self-management*)*,* and C_UK_ (*improved family coping and support*), though the latter with only a marginally significant loading. The second super profile conveyed a common focus on strengthening resilience by developing skills and insight within the individual or their family.

**Table 4:** Rotated component loadings for second-order PCA

Outcome priority profile	Practitioner Q-sort	Super profile	
		A_super_	B_super_
Super profile A: Reducing symptoms and enhancing well-being	A_UK+_ Reduced symptoms and enhanced well-being	0.96	0.15
	B_CL_ Reduced symptoms and enhanced well-being	0.73	0.26
	A_UK_- Reduced symptoms and enhanced well-being (polar opposite)	− 0.96	− 0.15
Super Profile B: Fostering resilience through the strengthening of skills, insights, or support	A_CL_ Strengthened identity and enhanced insight	0.90	0.03
	B_UK_ Improved individual coping and self-management	0.87	0.23
	C_UK_ Improved family coping and support	0.43	0.15
	% Variance explained	41.0	31.0
	Composite reliability coefficient	0.89	0.92

Descriptive exploratory analyses showed practitioners associated with super profile A_super_ (*reducing symptoms and enhancing well-being*) tended to be older (mean age = 46.6 years) than practitioners associated with super profile B_super_ (*fostering resilience through the strengthening of skills and insights*; mean age = 37.4 years), and had an average of seven additional years of professional experience in CAMH. With regards to professional roles, psychiatrists made up 36% of practitioners (*n* = 5) associated with profile A_super_ but only 5% of practitioners (*n* = 1) associated with super profile B_super_; in turn, psychotherapists accounted for only 7% (*n* = 1) in super profile A_super_, but 29% (*n* = 6) of practitioners in super profile B_super_. Practitioners associated with super profile A_super_ were more likely to use CBT approaches (62%) or pharmacotherapy (31%) than practitioners associated with super profile B_super_ (20% CBT; 5% pharmacotherapy), but less likely to use psychoanalytic or psychodynamic approaches (8% in A_super_ vs. 38% in B_super_).

## Discussion

This Q-study investigated differences in outcome priorities for treating youth depression among a qualitative sample of 27 mental health practitioners in the UK and 15 practitioners in Chile. In this sample, priorities were heterogenous with three major priority profiles emerging in the UK (i.e. *Reduced symptoms and enhanced well-being*; *improved individual coping and self-management*; *improved family coping and support*), and two profiles emerging in Chile (i.e. *strengthened identity and enhanced insight*; *symptom reduction and self-management*). In both contexts, practitioners divided into those focused on reducing symptoms and enhancing well-being, and those focused on building resilience by developing skills and resources within individuals or families. In line with this, second-order PCA identified two cross-cultural super profiles across the country-specific profiles: A_super_ (*reducing  symptoms and enhancing well-being*) and B_super_ (*fostering resilience through the strengthening of skills, insights, or support*).

A similar divide between practitioners has previously been identified by two UK-based Q-studies that focused on clinicians working with adults recovering from psychosis [41], and with adults recovering from borderline personality disorder [42]. The divide also aligns with two perspectives on recovery that are typically distinguished in the mental health recovery literature: a “clinical recovery concept” is thought to emphasize symptom remission and the restoration of functioning; a “personal recovery concept” is thought to be concerned with self-discovery and a search for meaning [66–69]. This literature considers that the “clinical recovery concept” is often adopted by clinicians, while the “personal recovery concept” is more closely associated with service user experiences. Our findings suggest that a relevant divide in perspectives also exists *among* practitioners.

Reducing risk of harm was a consensus outcome among practitioners in this study. This is in line with ICHOM recommendations whereby suicidal ideation and behaviour should be measured in all treatment-seeking youth with depression aged 10 years and older [32]. In addition, the first super profile’s focus on reduced depressive symptoms aligns with ICHOM’s recommendation to measure symptom severity in all youth presenting with depression. It also aligns with a strong focus on the measurement of symptom severity in published youth depression clinical trials [28, 32]. In contrast, the outcomes prioritized by the second practitioner super profile (i.e., improved coping skills, family functioning, parental support, personal growth) did not reach consensus as ICHOM-recommended core outcomes, and were rarely measured in youth depression treatment studies published between 2007 and 2017 [28]. Consequently, data and measurement recommendations for these outcomes are scarce.

Practitioners who prioritize these outcomes may find the existing evidence base less informative. They may also find commonly used measurement instruments less helpful in informing their clinical decision-making. The measurement recommendations made by core outcome sets such as those developed by ICHOM do not intend to be exhaustive. They present a *minimum* standard that can be complemented with additional outcomes that are considered important locally or in specific treatment contexts [70]. For example, add-on modules could be devised by associations or special interest groups for specific treatment approaches, or for use in specific cultural contexts. Developing measurement guidance for practitioners who prioritize outcomes beyond the symptom domain is important to ensure that measurement-based care can cater to their information needs. This will also require intensifying psychometric research into the validity, reliability and sensitivity to change of measures available in the domains of coping skills, family functioning, or personal growth to ensure that similar standards of rigour are applied as in the symptoms domain.

In a previous Q-study involving a sample of youth with lived experience of depression, we identified four outcome priority profiles. Youth, respectively, focused on “*relieving distress and experiencing a happier emotional state*”; “*learning to cope with cyclical distressing emotional states*”; “*understanding and processing distressing emotional states*”; and “*reduced interference of ongoing distressing emotional states with daily life*” [40]. The first youth profile conveyed a similar focus on symptom reduction and enhanced well-being as the practitioner super profile A_super_. The second youth profile conveyed a focus on acquiring practical coping skills and self-reliance, resembling that of UK practitioner profile B_UK_. The third youth profile focused on enhancing youth’s understanding of their difficulties and ability to process experiences internally—which bears some resemblance with Chilean profile A_CL_. In contrast, the fourth youth profile emphasized the reduction of functional impairment, with no direct equivalent among practitioner profiles. Similarly, the UK clinician profile C_UK_ that focused on improved family support had no equivalent among youth.

In clinical practice, mental health practitioners and youth may not adhere to the same outcome priority profiles. It may be helpful for pairs of practitioners and youth to establish and negotiate outcome priorities before engaging in shared decision-making about the best treatment options, and before agreeing on an outcome measurement strategy [71]. Where youth present with comorbidities, more complex conversations are likely needed to establish priority outcomes under consideration of transdiagnostic and condition-specific goals. A decision aid that presents different outcome domains might help with articulating and defining outcome priorities, as might idiographic patient reported outcome measures (I-PROMs) that enable youth to define change indicators around personal treatment goals [72].

In our descriptive, exploratory analysis of associations between practitioner characteristics and the two outcome priority super profiles, symptom-focused practitioners were more likely to use CBT than practitioners in the resilience-focused super profile, and tended to have more years of professional experience. Psychiatrists were overrepresented in this super profile, while psychotherapists were overrepresented in the resilience-focused profile. Being from Chile or the UK did not appear associated with priority profiles, although Chilean practitioners focused more strongly on outcomes related to personal growth (e.g., gaining a stronger sense of self), while UK practitioners focused more on CBT-type coping skills. This may reflect a stronger representation of CBT practitioners in the UK sample, and of psychoanalytic practitioners in the Chilean sample. This, in turn, might suggest that theoretical training is a more important determinant of outcome priorities than cultural background between the two contexts considered in this study, in line with suggestions whereby theoretical training influences judgments of what constitutes a “good outcome” [34–36]. Given the small sample size, these observations are tentative. Future research should explore cross-cultural nuances in outcome priorities in more depth, and also examine how specific terms and constructs are understood and defined locally [73]. Our findings underscore the importance of ensuring diversity in professional roles, treatment approaches, and cultural backgrounds in expert committees that aim to make globally applicable measurement recommendations [74].

### Strengths and limitations

To our knowledge, this is the first Q-study to investigate outcome priorities among mental health practitioners in relation to the treatment of youth depression. Q-methodology uniquely combines the mathematical principles of statistical analysis with the in-depth examination of subjectivity through the qualitative analysis of participant rationales [45]. This approach also brings limitations: our study used a qualitative convenience sample that may not be representative of the general population of mental health practitioners treating youth with depression in the UK and in Chile. Our study aimed to map the latent viewpoints regarding outcome priorities in this sample, but was not designed to inform generalizations about the distribution of these viewpoints in the wider population [45, 75]. We have made tentative observations about associations between practitioner characteristics and outcome priorities. These require further examination in larger, representative practitioner samples, for example using quantitative survey designs [76].

Due to administrative delays in the ethical approval process, the Chilean sample was considerably smaller than our recruitment target of 30 practitioners per country. Practitioners working in public mental health settings, and male practitioners were underrepresented in this sample, compared to the UK sample. This may have influenced outcome priorities, though additional research is needed to understand how outcome priorities may differ between private and public practice contexts.

It is critical to the content validity of a Q-study that the Q-set adequately represents the topic under study [77]. We used a rigorous multi-stage process for Q-set creation, but cannot rule out the possibility that an important outcome has been missed. During the post-sort interviews, two practitioners each suggested the Q-set could have covered romantic relationships, sleep, and the therapeutic alliance in addition to the 35 outcome descriptions that we did include. Other additional outcomes suggested by individual practitioners included resilience towards academic pressure, physical appearance, improvements in extended family relationships, and fostering support in young people’s wider social environment. However, no single outcome was consistently identified as missing from the Q-set.

This Q-study asked participants to sort their outcomes into a fixed quasi-normal distribution, with a limited number of slots provided under each rank. This facilitates sorting for participants, and analysis for the researcher. However, principal component analysis considers each item rank as equally valid and informative. If participants considered that several items were duplicative, and chose to assign one of these to a high rank of importance, while deprioritizing the other, the latter’s low position would be interpreted as face-valid, and possibly create a spurious association with participants who considered this outcome to be truly unimportant. Nevertheless, the components identified by this Q-study were theoretically plausible, and their interpretation drew closely on the post-sort interviews.

## Conclusion

Use of Q-methodology enabled the identification of three major outcome priority profiles among UK mental health practitioners, and two outcome priority profiles among Chilean mental health practitioners in relation to the treatment of youth depression. The context-specific profiles aggregated into two cross-cultural “super profiles” focused on enabling symptom reduction, and strengthening resilience resources, respectively. A practitioner focus on symptom reduction aligns with a long-standing focus on symptom measurement in youth depression treatment studies [28, 78, 79], and with recent outcome measurement recommendations for clinical practice [32]. In contrast, less outcome data and measurement guidance is available to practitioners who prioritize resilience-related outcomes as indicators of treatment success [31]. In the absence of measurement guidance that matches their outcome priorities, and in light of scarce reporting on these outcomes in the literature, practitioners prioritizing resilience outcomes may have less opportunity to engage in evidence-based practice, and may be more skeptical of engaging in measurement-based care. Future efforts to advance harmonized, person-centred, and high-quality outcome measurement in youth mental health should consider developing guidance around the measurement of a broader range of outcomes, beyond symptom reduction for both clinical practice and clinical trials.

### Supplementary Information

Below is the link to the electronic supplementary material.

Supplementary file1 (DOCX 37 KB)

## Data Availability

The datasets generated during the current study are not publicly available due to data privacy considerations.
