# Supplementary material for: What treatment outcomes matter in adolescent depression? A Q-study of priority profiles among mental health practitioners in the UK and Chile
Source: Eur Child Adolesc Psychiatry. 2023 Jan 31;33(1):151–66. doi: 10.1007/s00787-023-02140-9 (PMC10806045; doi:10.1007/s00787-023-02140-9)
Supplement: Supplementary file 1 — Supplementary file1 (DOCX 37 KB) [file 787_2023_2140_MOESM1_ESM.docx]

What Treatment Outcomes Matter Most in Adolescent Depression? A Q-Methodological Investigation of Priority Profiles Among Mental Health Practitioners in the UK and Chile

Karolin Rose Krause, Ana Calderón, Victor Gomez Pino, Julian Edbrooke-Childs, Bettina Moltrecht, Miranda Wolpert

**Supplement 1: Spanish Q-set**

**Table S1. Spanish Q-set Item Wording**

| # | Item wording |
| --- | --- |
| 1 | Que esté menos enojado/a y no se descontrole. |
| 2 | Que se sienta menos deprimido/a. |
| 3 | Que se sienta más feliz y disfrute más las cosas. |
| 4 | Que se sienta más querido/a. |
| 5 | Que participe menos en actividades que le puedan hacer daño (por ejemplo, autolesionarse, abusar de las drogas y alcohol). |
| 6 | Que sea más activo/a y participe activamente en las cosas. |
| 7 | Que conozca formas de controlar sus emociones. |
| 8 | Que tenga una mejor comprensión de sus sentimientos y pensamientos. |
| 9 | Que sea capaz de desafiar sus pensamientos negativos y enfrentar las situaciones de manera diferente. |
| 10 | Que sea más capaz de hacer cosas (por ejemplo, concentrarse, organizarse) |
| 11 | Que pueda hacer las mismas cosas que otros adolescentes hacen. |
| 12 | Que trabaje mejor en el colegio (por ejemplo, que esté más motivado/a y enfocado/a). |
| 13 | Que vaya al colegio más regularmente. |
| 14 | Que sea más sociable y capaz de estar con otras personas. |
| 15 | Que tenga una mayor confianza en sí mismo. |
| 16 | Que sea más capaz de defender sus necesidades y opiniones. |
| 17 | Que sea más independiente y asuma la responsabilidad por su propia vida. |
| 18 | Que pueda dar sentido a las cosas que le pasaron en el pasado, o que todavía le están pasando. |
| 19 | Que tenga una mejor idea de quién es y cómo ser él mismo con los demás. |
| 20 | Que se sienta más capaz de hablar sobre sus sentimientos y pensamientos. |
| 21 | Que se lleve mejor con su familia (por ejemplo, menos peleas, hablar más abiertamente, sentirse más apoyado/a) |
| 22 | Que se lleve mejor con sus amigos o haga nuevos amigos. |
| 23 | Que se lleve mejor con sus compañeros en el colegio (por ejemplo, que no le hagan bullying). |
| 24 | Que tenga un espacio donde alguien le escucha y se preocupa por él/ella. |
| 25 | Que tenga un espacio donde pueda expresar sus sentimientos. |
| 26 | Que tenga un espacio donde pueda hablar de cualquier cosa sin ser juzgado/a. |
| 27 | Que tenga un espacio para pensar sobre las cosas de manera diferente. |
| 28 | Que tenga más tranquilidad y estabilidad. |
| 29 | Que se sienta más optimista y positivo/a sobre la vida y el futuro. |
| 30 | Que se sienta con mejor salud física. |
| 31 | Que sea capaz de tener objetivos y hacer planes para el futuro. |
| 32 | Que sus padres se sientan menos estresados y preocupados por é/ella. |
| 33 | Que sus padres le entiendan más a él/ella y lo que le cuesta a su hijo/hija. |
| 34 | Que sus padres se sientan más capaces de apoyarle a su hijo/hija. |
| 35 | Que sus padres se sientan menos culpables. |
